# Identification of a Novel ERK5 (MAPK7) Inhibitor, MHJ-627, and Verification of Its Potent Anticancer Efficacy in Cervical Cancer HeLa Cells

**DOI:** 10.3390/cimb45070388

**Published:** 2023-07-24

**Authors:** Jeonghye Hwang, Hyejin Moon, Hakwon Kim, Ki-Young Kim

**Affiliations:** 1 Department of Genetics and Biotechnology, Kyung Hee University, Yongin 17104, Republic of Korea; hjh7956@khu.ac.kr; 2 Department of Applied Chemistry, Global Center for Pharmaceutical Ingredient Materials, Kyung Hee University, Yongin 17104, Republic of Korea; hjm6719@naver.com (H.M.); hwkim@khu.ac.kr (H.K.); 3 Graduate School of Biotechnology, Kyung Hee University, Yongin 17104, Republic of Korea

**Keywords:** ERK5, MAPK7, BMK1, ERK5 inhibitor, anticancer, oncotherapy, imidazolium

## Abstract

Extracellular signal-regulated kinase 5 (ERK5), a member of the mitogen-activated protein kinase (MAPK) family, is involved in key cellular processes. However, overexpression and upregulation of ERK5 have been reported in various cancers, and ERK5 is associated with almost every biological characteristic of cancer cells. Accordingly, ERK5 has become a novel target for the development of anticancer drugs as inhibition of ERK5 shows suppressive effects of the deleterious properties of cancer cells. Herein, we report the synthesis and identification of a novel ERK5 inhibitor, MHJ-627, and verify its potent anticancer efficacy in a yeast model and the cervical cancer HeLa cell line. MHJ-627 successfully inhibited the kinase activity of ERK5 (IC_50_: 0.91 μM) and promoted the mRNA expression of tumor suppressors and anti-metastatic genes. Moreover, we observed significant cancer cell death, accompanied by a reduction in mRNA levels of the cell proliferation marker, proliferating cell nuclear antigen (*PCNA*), following ERK5 inhibition due to MHJ-627 treatment. We expect this finding to serve as a lead compound for further identification of inhibitors for ERK5-directed novel approaches for oncotherapy with increased specificity.

## 1. Introduction

Extracellular signal-regulated kinase 5 (ERK5), also termed big mitogen-activated protein kinase 1 (BMK1) and mitogen-activated protein kinase 7 (MAPK7), belongs to the mitogen-activated protein kinase (MAPK) family, which mainly consists of four subfamilies in mammalian cells: ERK1/2, c-Jun-N-terminal kinases (JNK)1/2/3, p38α/β/γ/δ, and ERK5 [1]. In MAPK signaling cascades, three kinds of kinase are consecutively activated: a MAPK kinase kinase (MAPKKK), a MAPK kinase (MAPKK) and a MAP kinase (MAPK). In the ERK5 signaling pathway, MEKK2/3 are activated by various extracellular stimuli such as mitogens, cytokines, and cellular stresses, and they subsequently phosphorylate and activate MEK5 [2,3]. Once activated, MEK5 activates the apical kinase ERK5 by phosphorylating the T-E-Y motif in the activation loop within the ERK5 kinase domain [4,5]. Activated ERK5 then modulates a wide array of key cellular processes such as cell survival, proliferation, differentiation, angiogenesis, and apoptosis [6]. Structurally, the unique behavior of ERK5 among MAPK members is due to its extended C-terminal non-catalytic domain, which contains a transcriptional activation domain [7,8]. While other conventional MAP kinases transmit signals to downstream molecules mainly by phosphorylation, ERK5 can regulate downstream targets in two ways: direct substrate phosphorylation through its N-terminal kinase domain and transcriptional activation through autophosphorylation on its C-terminal non-kinase domain [9]. Thus, ERK5 is able to translocate to the nucleus and directly control gene expression by activating transcription factors [10].

With lines of accumulating research, overexpression and upregulation of ERK5 have been reported in various cancers, and ERK5 is widely implicated in the biological characteristics of cancer cells [11,12]. Moreover, ERK5 inhibition has been shown to suppress cancer cell proliferation, especially HeLa cells, and to induce tumor cell death in various tumor types [13,14,15,16]. Accordingly, ERK5 has emerged as a potential novel therapeutic target for overcoming malignancies and suppressing the deleterious actions of cancer cells [17].

However, to date, there has been a lack of high throughput screening systems to detect changes in ERK5 activity in animal cells due to its complex networks and cross-talk of signaling pathways [18]. Thus, we established a simple and time-saving yeast model system which could be utilized as a primary ERK5 inhibitor screening procedure to select putative ERK5 inhibitors among various candidate compounds we had (other compounds not mentioned), based on the well-established homologous pathway in yeast. Mpk1 (Slt2) in the CWI (Cell Wall Integrity) pathway that is functionally homologous to the ERK5 in humans is found in the yeast *Saccharomyces cerevisiae* [19]. It was demonstrated by Truman et al. that the expression of human ERK5 in Mpk1-defective yeasts is capable of rescuing diverse phenotypes attributable to the loss of native Mpk1 and therefore yeast Mpk1 is a functional homologue of human ERK5 [19]. The C-terminal domain of Mpk1 also possesses a transcriptional activating potential like ERK5, not to mention the striking sequence similarity within the N-terminal domain (49.7%) [19,20]. Since it is known that Mpk1 activates Rlm1 transcription factor by directly phosphorylating it and Rlm1 subsequently activates the transcription of *MLP1*, the decrease in *MLP1* expression shown by the β-galactosidase reporter can be interpreted as inhibited catalytic activity of Mpk1 (ERK5 homologue). This model system enables us to easily observe the alteration in Mpk1 activity using the *MLP1-lacZ* reporter plasmid. Moreover, there are two types of transcriptional regulatory pathways of ERK5, one reliant on the kinase domain and the other on the transcriptional activation domain. Since it is demonstrated by Jung et al. that Mpk1-Rlm1-*MLP1* pathway is mediated by the catalytic action of Mpk1, this model system makes it easier to achieve our goal to develop a kinase inhibitor of ERK5 [21]. 

Therefore, we ultimately aimed to develop a potential anticancer drug candidate for ERK5 inhibition through a series of experiments in a yeast model and the cervical cancer HeLa cell line. We report the synthesis and identification of a novel ERK5 inhibitor, MHJ-627, and verify its potent anticancer efficacy.

## 2. Materials and Methods

### 2.1. Instruments and Chemicals

All chemical reagents were purchased from Acros Organics (Brookline, MA, USA), Alfa Aesar (Haverhill, MA, USA), Sigma-Aldrich (St. Louis, MO, USA) or Tokyo Chemical Industry (Tokyo, Japan) and were used as received. The progress of reactions was monitored through thin-layer chromatography (TLC, silica gel 60 F254; Merck, Darmstadt, Germany). Melting points (m.p.) were determined on a Barnstead Electrothermal 9100 instrument and were uncorrected. ^1^H and ^13^C NMR spectra were recorded on a JEOL JNM-ECZ400S (Tokyo, Japan). NMR solvent was purchased from Cambridge Isotope Laboratories, Inc. (Andover, MA, USA) and spectra are referenced relative to the chemical shift of tetramethylsilane (TMS) as an internal standard. Chemical shifts (*δ*) are reported in parts-per-million (ppm), and coupling constants (*J*) are reported in Hertz (Hz). High-resolution mass spectroscopy was performed with a JEOL JMS-700 mass spectrometer. 1-(1,4-Bis(isopentyloxy)naphthalen-2-yl)-2-bromoethanone (2) and 1-isopentyl-1*H*-benzo[*d*]imidazole (3) were prepared as previously described [22,23].

Synthesis of 3-(2-(1,4-bis(isopentyloxy)naphthalen-2-yl)-2-oxoethyl)-1-isopentyl-1*H*-benzo[*d*]imidazol-3-ium bromide, MHJ-627: a solution of 1-(1,4-bis(isopentyloxy)naphthalen-2-yl)-2-bromoethanone (2, 0.10 g, 0.24 mml, 1.0 eq) and 1-isopentyl-1*H*-benzo[*d*]imidazole (3, 0.045 g, 0.24 mmol, 1.0 eq) in acetonitrile (4.8 mL, 0.05 M) was stirred at reflux for 24 h. After the reaction was complete, the product was concentrated in vacuo and recrystallized in ether to yield MHJ-627 as an ivory solid. Yield: 80%. m.p.: 195.6–197.3 °C. ^1^H NMR (400 MHz, (CD_3_)_2_SO) δ (ppm): 0.97–1.00 (m, 18H), 1.61–1.71 (m, 1H), 1.75–1.80 (m, 2H), 1.84–1.97 (m, 6H), 4.21 (t, *J* = 6.4 Hz, 2H), 4.25 (t, *J* = 6.4 Hz, 2H), 4.65 (t, *J* = 7.6 Hz, 2H), 6.26 (s, 2H), 7.23 (s, 1H), 7.67–7.81 (m, 4H), 8.09 (dd, *J* = 1.2, 7.6 Hz, 1H), 8.17 (dd, *J* = 1.2, 7.6 Hz, 1 H), 8.19–8.28 (m, 2H), and 9.82 (s, 1H). ^13^C NMR (100 MHz, (CD_3_)_2_SO) δ (ppm): 22.10, 22.40, 22.60, 24.59, 24.78, 24.98, 37.14, 37.25, 38.48, 45.23, 55.83, 66.55, 75.51, 102.06, 113.67, 114.41, 122.30, 123.43, 123.68, 126.52, 126.68, 127.83, 128.25, 129.00, 129.05, 130.65, 131.96, 143.39, 150.58, 151.41, and 190.94. HRMS (FAB+ mode) m/z Calcd. for C_34_H_45_N_2_O_3_ [M-Br]+ 529.3430, found 529.3433.

A commercialized ERK5 inhibitor, XMD8-92 (S7525), and a MEK1/2 inhibitor, U0126 (S1102), were purchased from Selleck Chemicals (Houston, TX, USA). 

### 2.2. Yeast Strains, Plasmids, Growth Conditions, and Transformation

The *S. cerevisiae* strain BY4742 was grown in a YEPD medium containing 2% Bacto peptone, 1% Bacto yeast extract, and 2% glucose at 30 °C in a shaking incubator [24]. *Escherichia coli* DH5α was used to distribute the plasmids. For selective growth, they were grown in a Luria Bertani (LB) medium containing 1% Bacto-tryptone, 0.5% Bacto-yeast extract, 1% NaCl, and 100 μg/mL Ampicillin at 37 °C in a shaking incubator. The plasmid used to transform the yeast contains an *MLP1* promoter followed by *lacZ*, which makes it possible to detect the expression level of *MLP1* through β-galactosidase expression [24,25,26]. Yeasts were transformed with *MLP1*-*lacZ*-containing plasmids using the standard lithium acetate-PEG method. Yeast transformants were cultured in a synthetic defined (SD) medium without uracil (SD-Ura) at 23 °C in a shaking incubator for 18 h until the exponential growth phase and then moved to a YEPD medium and cultured for 18 h to produce enough cells for the experiment [27]. For the ONPG assay, yeast cells were adjusted to OD_600_ = 1.0 with YEPD medium.

### 2.3. Animal Cell Lines and Culture

Human cervical cancer cell line HeLa cells (Korean Cell Line Bank, Seoul, Republic of Korea) were selected since they are commonly used in the study of ERK5 due to their ability to provide a clear observation of ERK5 activity [28,29]. It is known that negative regulation of ERK5 induces apoptosis in HeLa cells since ERK5 activity is necessary for survival of HeLa cells [16]. Cells were routinely cultured in Dulbecco’s Modified Eagle’s Medium (DMEM) high glucose, supplemented with 10% (*vol*/*vol*) fetal bovine serum (FBS) and 1% (*vol*/*vol*) penicillin/streptomycin. HeLa cells were grown at 37 °C in a humidified incubator with 5% CO_2_ [30]. 

### 2.4. β-Galactosidase Reporter Assay

Yeast cells bearing *MLP1*-*lacZ* reporter plasmids were prepared as described above. For this, 3 mL of cells (OD_600_ = 1.0) were treated with 15 μL of compounds. Yeast cells were prepped via centrifugation and resuspended in 250 μL of breaking buffer (100 mM Tris-HCl pH = 8, 1 mM dithiothreitol, and 20% glycerol), with 100 μL of glass beads of 0.4–0.6 mm in diameter [31]. Yeast cells were homogenized via a bead beater to extract proteins. After 6 cycles of bead beating, samples were clarified via centrifugation at 12,000 RPM for 15 min at 4 °C. The Bradford method was used to measure protein concentration. Here, 100 μL of protein extracts containing 15 ug of proteins were mixed with 900 μL of Z buffer (60 mM Na_2_HPO_4_∙7H_2_O, 40 mM NaH_2_PO_4_∙H_2_O, 10 mM KCl, 1 mM MgSO_4_∙7H_2_O, 50 mM 2-Mercaptoethanol, and pH = 7.0). After 5 min at 28 °C, 200 μL of O-nitrophenyl-β-D-galactopyranoside (ONPG) solution (4 mg/mL in Z buffer) was added. The reaction was conducted at 28 °C in a water bath for 3 h until the mixture obtained a pale-yellow color. The reaction was terminated by adding 500 μL of Na_2_CO_3_ solution. To measure the degree of ONPG hydrolysis by β-galactosidase, optical density was measured at 420 nm using a spectrophotometer [32]. All the procedures are based on Rose and Botstein’s method [26,33,34]. Miller unit was calculated as follows.

$$\frac{{OD}_{420}\;\times 1.7}{0.0045\times \mathrm{protein}\;\mathrm{concentration}\;(\mathrm{mg}/\mathrm{mL})\times \mathrm{protein}\;\mathrm{extract}\;\mathrm{volume}\;(\mathrm{mL})\times \mathrm{time}\;(m)}$$

### 2.5. In Vitro Kinase Assay

An in vitro kinase assay was conducted to determine the inhibition of ERK5 kinase activity caused by MHJ-627 at concentrations of 5 μM, 1 μM, 0.1 μM, and 0 μM. The kinase assay was performed using Z’-LYTE™ Kinase Assay Kit—Ser/Thr 4 Peptide (PV3177; Thermo Fisher Scientific, Waltham, MA, USA) following the manufacturer’s instruction. For this, 9 ng of ERK5 (ab126913; Abcam, Eugene, OR, USA) was used per one kinase reaction, and 100 μM ATP was used to drive the kinase reaction [35]. Fluorescence intensity was detected with a Varioskan™ LUX multimode microplate reader (VL0000D0; Thermo Fisher Scientific, Waltham, MA, USA).

### 2.6. Transient Transfection and qRT-PCR-Based Luciferase Reporter Assay

To measure the activity of AP-1, which is activated by ERK5, HeLa cells were transfected with pGL4.44 plasmid [*luc2P*/AP1 RE/Hygro] containing six copies of an AP-1 response element (AP1 RE), which drives transcription of the luciferase reporter gene *luc2P* (Photinus pyralis), using a LipofectamineTM 3000 reagent (Invitrogen, Waltham, MA, USA) according to the manufacturer’s protocol [36]. After 24 h of transfection, the cells were seeded at a density of 3 × 10^5^ cells per well in a 6-well plate. After 24 h, the cells were treated with MHJ-627 at concentrations of 5 μM, 1 μM, 0.1 μM, and 0 μM, as well as with XMD8-92 (positive control) at a concentration of 5 μM. To measure the mRNA expression level of luciferase, quantitative real-time PCR was conducted.

### 2.7. Quantitative Real-Time PCR Analysis

HeLa cells were seeded at a density of 3.0 × 10^5^ cells per well of 6-well plates in 2 mL of serum-containing DMEM and were further cultured for 24 h for attachment. Then, various concentration (5 μM, 1 μM, and 0.1 μM) of MHJ-627 dissolved in 2 mL of serum-free DMEM were added, and cells were further cultured for 24 h. After 24 h of treatment, total RNA was isolated using a Trizol reagent (Thermo Fisher Scientific, Waltham, MA, USA) according to the manufacturer’s protocol and reverse-transcribed to cDNA [37]. qRT-PCR was carried out using 2X SybrGreen Real-Time PCR Master Mix (Biofact, Daejeon, Republic of Korea). A housekeeping gene, *GAPDH*, served as an endogenous control [38]. Sequences of the primers used are listed in Table 1. 2^−ΔΔCq^ was calculated in duplicate, and an average of the two values was used to analyze expression of the genes [39].

### 2.8. Western Blot Analysis

HeLa cells were seeded at a density of 3.0 × 10^5^ cells per well of 6-well plates in 2 mL of serum-containing DMEM and were further cultured for 24 h for attachment. Then, various concentrations (5 μM, 1 μM, 0.1 μM, and 0 μM) of MHJ-627 dissolved in 2 mL of serum-free DMEM were applied to cells and further cultured for 24 h. After 24 h treatment, cells were lysed in radio-immunoprecipitation assay (RIPA) buffer containing 150 mM sodium chloride, 1% Triton X-100, 0.5% sodium deoxycholate, 0.1% sodium dodecyl sulfate (SDS), 50 mM Tris (pH 8.0), and a complete protease inhibitor cocktail (BIOMAX, Seoul, Republic of Korea). Protein concentration was determined using the BCA protein assay kit (TaKaRa, San Jose, CA, USA) according to the manufacturer’s protocol. An equal amount of protein (10 μg/lane) was separated using sodium dodecyl sulfate-polyacrylamide gel electrophoresis (SDS-PAGE), transferred to a polyvinylidene difluoride (PVDF) membrane, and blocked with 5% BSA and 5% skim milk in a TBST buffer (20 mM Tris-HCl, 150 mM NaCl, and 0.1% Tween 20, pH 7.6) [37]. The membranes were probed with primary antibodies against GAPDH (sc-25778), ERK5 (sc-398015), and phospho-ERK5 (sc-135760) (Santa Cruz Biotechnology, Inc., Dallas, TX, USA) at 4 °C overnight. Membranes were then incubated with secondary antibodies for one hour at room temperature. Protein bands were developed with an ECL reagent and detected using a UVITEC imaging system equipment (UVITEC, Cambridge, UK) [40,41]. Relative protein expression from the Western blot data was determined using ImageJ.

### 2.9. Cytotoxicity Assay

Cytotoxicity, the ability of compounds to kill cancer cells, was evaluated via methylthiazol tetrazolium (MTT) assay. HeLa cells were seeded at a density of 1.0 × 10^4^ cells per well of 96-well plates in 100 μL serum-containing DMEM and were further cultured for 24 h for settlement as described during cell culture [42,43]. Subsequently, 100 μL of serum-free DMEM compounds with various concentrations was added to each well, and cells received 24 h compound exposures. The reason for serum starvation was to eliminate the possibility of serum affecting the results of the assay and to only observe the effects of the treated compounds [44,45,46,47]. MHJ-627, at concentrations of 100 μM, 50 μM, 10 μM, 5 μM, 1 μM, 0.1 μM, and 0 μM, was added to the cells. Compounds were dissolved in 100% dimethyl sulfoxide (DMSO) at the original concentration of 10 mM. In order to prevent the dilution of DMSO from interfering with the results, dilution proceeded while maintaining the same percentage of DMSO in the treatment. After 24 h of compound exposures, MTT solution (5 mg/mL) was diluted 10 times in serum-free DMEM and then added to the wells after suction. Then, the plate was further maintained at 37 °C in the incubator for 3 h. Briefly, 100 μL of DMSO was added to each well after suction in order to dissolve the formazan crystals, and the plate was wrapped in aluminum foil to avoid light and gently shaken on an orbital shaker for 30 more minutes [48]. Absorption values at 540 nm and 570 nm were measured via a microplate spectrophotometer (BioTek Instruments, Winooski, VT, USA). Survival of untreated cells was regarded as a negative control and set as 100%. Then, survival of treated cells was calculated as a percentage of negative control [49]. As MTT showed that most of the cells were dead at 10 μM of MHJ-627, 5 μM was set as the maximum concentration in other experiments.

### 2.10. Statistical Analysis

All of the experiments were performed in duplicate and independently repeated at least 3 times. All the data are presented as the mean ± standard deviation (SD). Statistically significant differences were analyzed using two-tailed *t* test when only two groups were compared and one-way ANOVA with Dunnett’s post hoc test using 0 μM as a control when more than two groups were compared [50]. *p* < 0.05 was considered statistically significant. All statistical analyses were conducted using GraphPad Prism software program version 5.0 (Graphpad Software, La Jolla, CA, USA).

## 3. Results and Discussion

### 3.1. MHJ-627 Compound Synthesis

**Figure 1:**
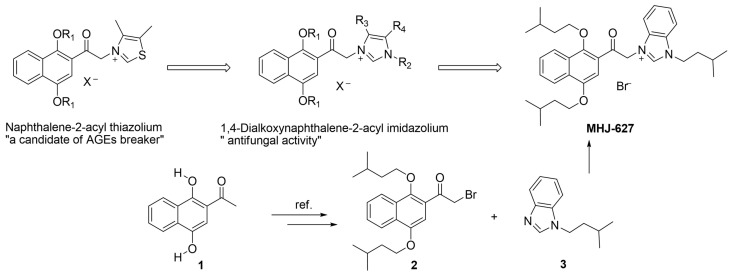
Design and synthesis of the new 1,4-dialkoxynaphthalen-2-acyl imidazolium salt, MHJ-627.

In our previous study (Figure 1), we synthesized various naphthalene-2-acyl thiazolium salts by combining the structures of 1,4-dialkoxynaphthalene and thiazole and evaluated their potential as AGE (advanced glycation end products) breakers [51]. The 1,4-dialkoxynaphthalene moiety played a significant role in their pharmacological activity. Subsequently, we replaced thiazole with an imidazole ring to produce 1,4-dialkoxynaphthalene-2-acyl imidazolium salt derivatives, which exhibited antifungal activity [22]. We further confirmed that combination with the 1,4-dialkoxynaphthalene moiety served as a good pharmacophore. After screening the activity of several 1,4-dialkoxynapthalene imidazolium salts, it was confirmed that MHJ-627 is a potent ERK5 inhibitor (Figure 1). 

The synthesis of MHJ-627 was carried out as follows: a key intermediate acyl bromide 2 was synthesized from a commercially available starting compound 1 using a known process [22] and subsequently reacted with benzimidazole 3 to produce the desired compound, MHJ-627.

### 3.2. MHJ-627 Suppressed the Catalytic Activity of Mpk1 to Activate Rlm1 Transcription Factor and Attenuated the Expression of MLP1

**Figure 2:**
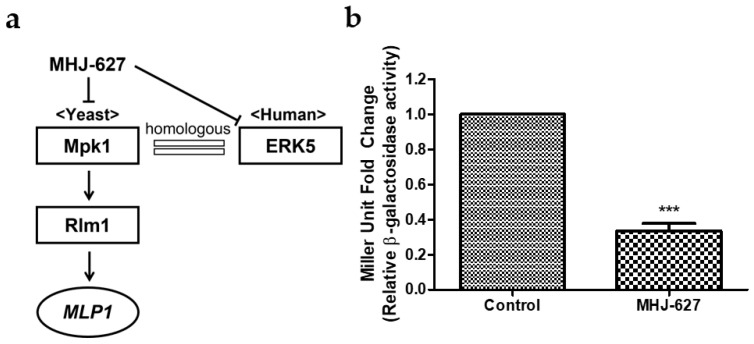
MHJ-627 suppressed the kinase activity of Mpk1 and attenuated *MLP1* expression in an *S. cerevisiae* model. (**a**) Schematic representation of Mpk1 regulation in the *S. cerevisiae* model system, which is functionally homologous to the human ERK5. Inactivation of Mpk1 activity results in downregulated transcriptional activity of Rlm1 transcription factor and subsequent decrease in *MLP1* expression; (**b**) effect of MHJ-627 on expression of *MLP1* measured by β-galactosidase activity. Yeasts were transformed with *MLP1*-*lacZ* reporter plasmid and treated with 15 μL of DMSO (control) and MHJ-627 in 3 mL of media. The data were calibrated to the control value (DMSO control = 1). Data are presented as mean ± SD. Each experiment was performed in duplicate and repeated at least three times. Two-tailed unpaired Student’s *t* test (*** *p* < 0.001) was used for significance.

As a primary putative ERK5 inhibitor screening procedure among various candidate compounds (other compounds not mentioned), we evaluated the ability of MHJ-627 to reduce the kinase activity of Mpk1, a functional homologue of human ERK5 [19], by examining the expression of *MLP1*, a target gene of Mpk1, through a transcriptional reporter assay using an *MLP1*-*lacZ* reporter plasmid in a yeast model system [24]. It is known that Rlm1 transcription factor activated by the kinase activity of Mpk1 promotes the transcription of *MLP1* (Figure 2a) [24]. MHJ-627 significantly suppressed *MLP1* expression by 66% compared to the control treated with DMSO only (Figure 2b). Since Rlm1 regulation is already demonstrated to be dependent on kinase activity of Mpk1 [21,24], this result implies that MHJ-627 impaired the kinase activity of Mpk1 to phosphorylate Rlm1 transcription factor and consequently inhibited *MLP1* expression, suggesting that MHJ-627 may also inhibit the kinase activity of human ERK5.

### 3.3. MHJ-627 Inhibited the Kinase Activity of Human ERK5 In Vitro

**Figure 3:**
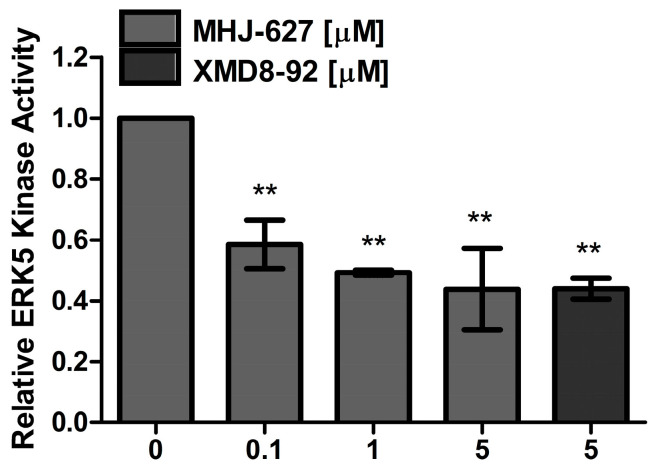
MHJ-627 reduced the kinase activity of human ERK5 in vitro. Relative ERK5 kinase activity following MHJ-627 treatment was measured via in vitro kinase assay. Kinase activity of ERK5 was reduced dose-dependently, supporting ERK5-inhibitory activity of MHJ-627 in vitro. Relative ERK5 kinase activity of the 0 μM control was set as 1. Data are presented as mean ± SD. Each experiment was performed in duplicate and repeated at least three times. One-way ANOVA (** *p* < 0.01) was used for significance. All values were compared to the 0 μM control value to determine the significance.

To further verify the capability of MHJ-627 to inhibit the kinase activity of human ERK5, a FRET-based in vitro kinase assay was carried out [52]. Relative kinase activity of ERK5 dropped to 0.58 at 0.1 μM, 0.49 at 1 μM, and 0.44 at 5 μM, which means MHJ-627 exhibited inhibitory activity by 42% at 0.1 μM, 51% at 1 μM, and 56% at 5 μM, respectively (Figure 3). This dose-dependent decrease in kinase activity according to the concentration of MHJ-627 shows that MHJ-627 also impairs the kinase activity of human ERK5 (IC_50_: 0.91 μM), as we expected from the previous yeast screening. XMD8-92, a commercialized ERK5 inhibitor, was used as a positive control and showed an inhibition rate of 56% at 5 μM, which means that MHJ-627 and XMD8-92 exhibit similar inhibitory activity at 5 μM in vitro [33,52]. Since we confirmed that most of the cells were severely affected at 10 μM of MHJ-627 in the MTT assay, 5 μM was set as the maximum concentration in this assay.

### 3.4. MHJ-627 Suppressed the Activity of ERK5 and Impaired AP-1 Activity in HeLa Cells

**Figure 4:**
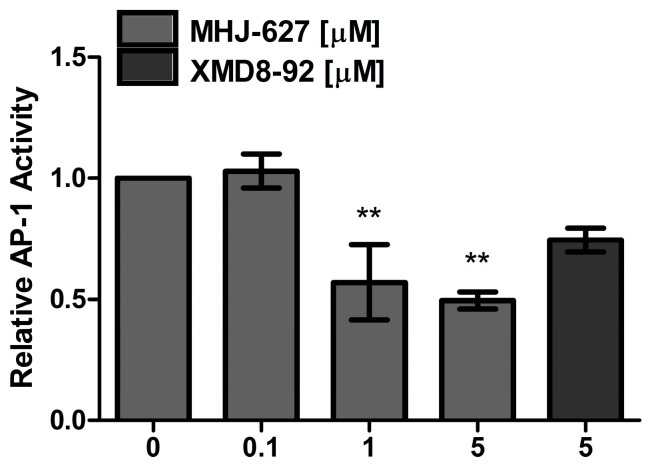
MHJ-627 suppressed ERK5 kinase activity to activate AP-1 transcription factor. To determine the ability of ERK5 to activate the transcription factor AP-1, luciferase reporter plasmid was transformed into HeLa cells and qRT-PCR was conducted to measure the mRNA level of luciferase after 24 h compound treatment. There was a decrease in luciferase mRNA levels, indicating reduced activity of AP-1 possibly caused by suppressed activity of ERK5 to activate AP-1. Relative AP-1 activity of the 0 μM control was set as 1. Data are presented as mean ± SD. Each experiment was performed in duplicate and repeated at least three times. One-way ANOVA (** *p* < 0.01) was used for significance. All values were compared to the 0 μM control value to determine the significance.

To further examine the ability of MHJ-627 to inhibit the kinase activity of human ERK5 in cells, activation of activator protein-1 (AP-1), a downstream transcription factor of ERK5, was measured via a luciferase reporter [36]. HeLa cells were transfected with a plasmid bearing an AP-1 response element followed by a luciferase reporter gene and the mRNA level of luciferase was measured using quantitative real-time PCR (qRT-PCR). As the AP-1 transcription factor is a downstream target of ERK5, even though it is also a downstream of ERK1/2, it is often used to evaluate the alteration in ERK5 activity in cells [53]. Since ERK5 is often dysregulated in cancers, AP-1 is also found in a hyperactivated form in tumor cells [54,55]. The mRNA of luciferase transcribed by the AP-1 transcription factor decreased in a dose-dependent manner (Figure 4), signifying hindered AP-1 activation by ERK5 following MHJ-627 and XMD8-92 treatment [56]. This result suggests that MHJ-627 successfully inhibits ERK5 both in vitro and at the cell level. 

### 3.5. ERK5 Inhibition by MHJ-627 Modified the mRNA Expression of Genes Regulated by ERK5

**Figure 5:**
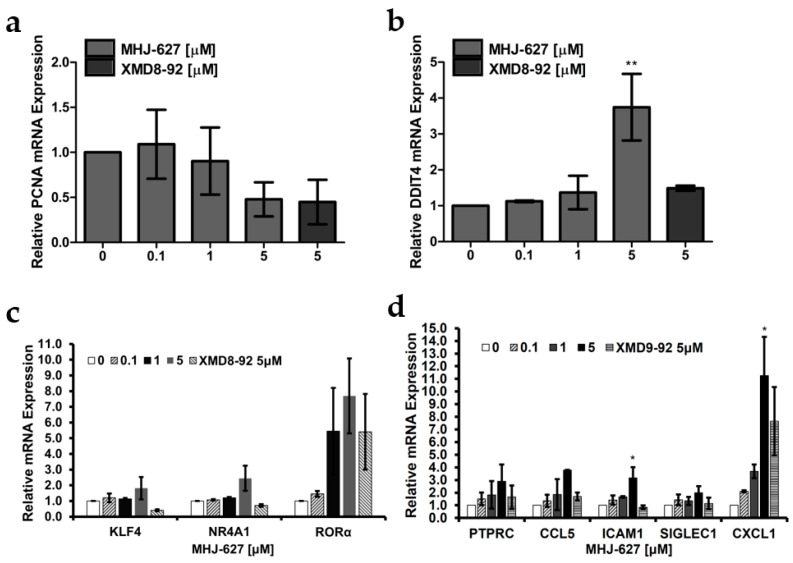
Alteration in mRNA expression pattern of the genes influenced by ERK5 after MHJ-627 treatment. (**a**) Decrease in mRNA expression of *PCNA*, which is a cell proliferation marker; (**b**) increase in mRNA expression of *DDIT4*, which is reported to increase when ERK5 is inhibited; (**c**) increase in mRNA expression of genes that encode transcription factors; (**d**) increase in mRNA expression of genes that encode immune-related proteins. Relative mRNA expression of genes influenced by ERK5 was measured via qRT-PCR analysis after 24 h compound treatment in HeLa cells. Relative mRNA expression of the 0 μM control was set as 1. Data are presented as mean ± SD. Each experiment was performed in duplicate and repeated at least three times. One-way ANOVA (* *p* < 0.05, ** *p* < 0.01) was used for significance. All values were compared to the 0 μM control value to determine the significance.

qRT-PCR was performed to assess the expression of genes that are previously reported to be regulated or influenced by ERK5 in the gene expression analyses of ERK5 signaling though they are not the established direct targets of ERK5 [57]. As previously reported, downregulation of proliferating cell nuclear antigen (PCNA) expression is a known effect of ERK5 inhibition or ablation. Consistently, we observed a decrease in mRNA expression of *PCNA*, which is involved in DNA replication and repair machinery (Figure 5a) [58,59,60]. In contrast, as illustrated by previous literature, the mRNA level of DNA damage-inducible transcript 4 (*DDIT4*), which acts as a negative regulator of the mammalian target of rapamycin (mTOR) pathway, was elevated (Figure 5b) [57,61]. Furthermore, we observed increases in mRNA expression of the genes that can be categorized into two groups based on the function of the proteins they encode: transcription factors and immune-related proteins. The mRNA expression of KLF transcription factor 4 (*KLF4*), nuclear receptor subfamily 4 group A member 1 (*NR4A1*) and retinoic acid receptor-related orphan receptor-alpha (*RORα*), which act as transcription factors, was upregulated (Figure 5c). There was an increase in mRNA expression of protein tyrosine phosphatase receptor type C (*PTPRC*), C-C motif chemokine ligand 5 (*CCL5*), intercellular adhesion molecule 1 (*ICAM1*), sialic acid binding Ig like lectin 1 (*SIGLEC1*), and C-X-C motif chemokine ligand 1 (*CXCL1*), which all are related to immunity (Figure 5d). Since an activation of immune cells and immune responses following ERK5 inhibition has been reported, we speculate that this increase in expression is due to a feedback loop of the signal transduction pathway [11]. The decrease in *PCNA* mRNA and increase in *DDIT4* and *CXCL1* mRNA, which have been reported to occur when ERK5 is inhibited, are evidence that MHJ-627 effectively targets ERK5 [58]. 

Especially, PCNA, which is distinctly considered a cell proliferation marker due to its accumulation in late G1 and S phases, is strongly suggested to be involved in cell survival and tumorigenesis [62,63]. Considering previous knowledge that the degradation of PCNA inhibits cancer proliferation in vitro and in vivo, a dose-dependent decrease in *PCNA* mRNA levels may be indicative of the anticancer efficacy of MHJ-627 [64]. Therefore, in future study, we will conduct in-depth study on how ERK5 inhibition downregulates PCNA and verify if PCNA could be a direct target of ERK5. *KLF4* is suggested to act as a tumor suppressor, and its expression is often downregulated in some types of cancer, including cervical cancer, colorectal cancer, and lung cancer [65,66,67]. Particularly, in cervical cancer, previous study has shown the inactivation of *KLF4* as a tumor suppressor [68]. Similarly, *RORα* is a potential tumor suppressor, and its downregulation, which is related to tumor progression, is often observed in cancers [69,70]. Nuclear receptor 4A1 (*NR4A1*) is proposed to be downregulated in metastatic tumors and to play a protective role against metastasis [71,72]. Taken together, these results suggest that MHJ-627-induced ERK5 inhibition contributes to establishing a suitable environment to overcome malignancies by promoting the expression of some tumor suppressors and anti-metastatic genes which we assumed to be an outcome of targeting overexpressed ERK5 in cancers. However, in the cases of *KLF4*, *NR4A1*, and *ICAM1*, the trend of alteration in mRNA expression when treated with MHJ-627 was different from the positive control, increasing in the MHJ-627 treatment while decreasing in the positive control treatment [73]. Since the ERK5-inhibitory effect of MHJ-627 was already demonstrated via in vitro kinase assay in Figure 3, this result indicates that the mechanism governing ERK5 inhibition of these compounds may be somewhat different and needs to be further investigated in a follow-up study to identify the precise mechanism of MHJ-627’s inhibition of ERK5 activity.

### 3.6. MHJ-627 Paradoxically Increased ERK5 Expression Possibly due to the Stimulatory Crosstalk of the ERK1/2 Pathway

**Figure 6:**
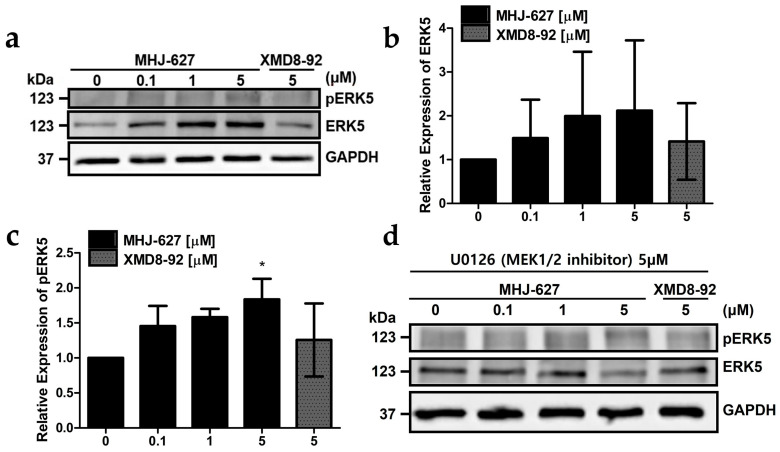
MHJ-627 paradoxically upregulated the expression and phosphorylation of ERK5, possibly due to the stimulatory crosstalk of the ERK1/2 pathway. (**a**) Western blot image depicting the elevations in ERK5 protein expression and phosphorylation. Effect of MHJ-627 on the protein expression and phosphorylation of ERK5 was measured via Western blot analysis after HeLa cells were treated with compounds for 24 h; (**b**) quantitation of Western blot showing a paradoxical increase in ERK5 expression; (**c**) quantitation of Western blot showing a trend of increase in ERK5 phosphorylation; (**d**) the increase in ERK5 expression and phosphorylation was due to the compensatory action of ERK1/2. GAPDH was used as a loading control. Relative protein expression of the 0 μM control was set as 1. Western blot data were quantified using ImageJ software. Data are presented as mean ± SD. Each experiment was performed in duplicate and repeated at least three times. One-way ANOVA (* *p* < 0.05) was used for significance. All values were compared to the 0 μM control value to determine the significance.

To determine whether MHJ-627 affects the protein expression levels of ERK5 and pERK5, Western blot analysis was conducted. MHJ-627 paradoxically appeared to elevate ERK5 expression and phosphorylation, and so did the positive control, XMD8-92 (Figure 6a–c). However, even though the protein expression and phosphorylation of ERK5 increased, previous experimental results from Figure 3 and Figure 4 have already shown that the actual activity of ERK5 was successfully inhibited as expected. 

Since crosstalk and feedback loop mechanisms of other signaling pathways have been suggested as the most challenging problem in developing kinase inhibitors, we assumed that the elevations in ERK5 expression and phosphorylation may be attributed partly to the stimulatory crosstalk and compensatory action of the PI3K-AKT pathway or the ERK1/2 pathway [74,75]. Therefore, we examined the effect of the ERK1/2 pathway by treatment with 5 μM of an MEK1/2 inhibitor, U0126, which inhibits the activation of ERK1/2, together with various concentrations of MHJ-627 [76]. As expected, protein expression and phosphorylation of ERK5 among the lanes showed no difference (Figure 6d), suggesting that the previous increase in expression was due to the compensatory action of the ERK1/2 pathway, at least in part. Nevertheless, the precise mechanism is still unknown and is yet to be identified.

### 3.7. MHJ-627 Showed Anti-Proliferative Effect in the Human Cervical Cancer HeLa Cells

**Figure 7:**
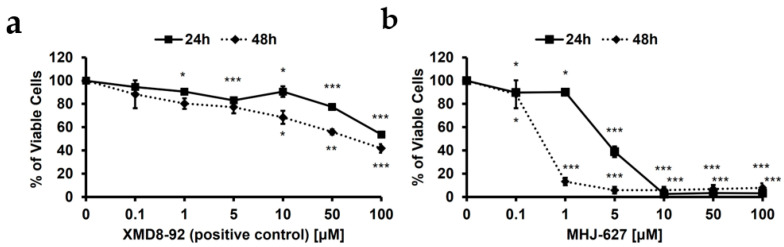
MHJ-627 had an anti-proliferative effect through inhibition of ERK5. (**a**) Effect of XMD8-92 on viability of HeLa cells showed that inhibition of ERK5 exhibited an anti-proliferative effect for HeLa cells; (**b**) effect of MHJ-627 on viability of HeLa cells showed its potent anti-proliferative efficacy. Cell viability was determined via MTT assay after 24 h and 48 h compound treatments with indicated concentration. XMD8-92 was used as a positive control. MHJ-627 showed higher cytotoxicity compared to the positive control, suggesting its potent anticancer efficacy and ERK5-inhibitory activity since ERK5 activity is necessary for the survival of HeLa cells. (0 μM control = 100%). Data are presented as mean ± SD. Each experiment was performed in duplicate and repeated at least three times. One-way ANOVA (* *p* < 0.05, ** *p* < 0.01, *** *p* < 0.001) was used for significance. All values were compared to the 0 μM control value to determine the significance.

To measure the anticancer efficacy of MHJ-627, an MTT assay was conducted. HeLa cells were treated with the indicated concentration of MHJ-627 for 24 h and 48 h. XMD8-92 was used as a positive control. HeLa cells treated with XMD8-92 showed a significant decline in cell viability and showed an anti-proliferative effect of 16.9% after 24 h and of 22.7% after 48 h at 5 μM treatment, providing evidence for the possible anticancer efficacy of ERK5 inhibition (Figure 7a). The viability of HeLa cells significantly decreased in a dose-dependent manner after MHJ-627 treatment (Figure 7b). Especially, MHJ-627 exhibited anti-proliferative effect of 61% after 24 h (IC_50_: 2.45 μM) and 94.2% after 48 h at 5 μM treatment. Almost every cancer was severely affected at a concentration of 10 μM in both the 24 h and 48 h treatments. The fact that MHJ-627 significantly exhibited higher cytotoxicity in HeLa cells confirms the higher anticancer efficacy of MHJ-627, which possibly resulted from the stronger ERK5-inhibitory activity, since negative regulation of ERK5 is known to induce apoptosis in HeLa cells [16]. This result casts a new light on the promise that MHJ-627 may serve as a more potent ERK5 inhibitor than the ones previously identified. 

## 4. Conclusions

ERK5 is a rising therapeutic target to combat cancer since its overexpression and dysregulation have been reported in various types of cancer [11,12]. However, despite its pivotal involvement in tumorigenesis, most previous works have focused on ERK1/2. In this study, we synthesized a novel ERK5 inhibitor, MHJ-627, and verified its potent anticancer property in the cervical cancer HeLa cells. MHJ-627 successfully impaired the kinase activity of ERK5 to produce significant anticancer efficacy accompanying upregulation of tumor suppressors and anti-metastatic genes, suggesting MHJ-627 as a promising ERK5 inhibitor.

There is no doubt that inhibition of ERK5 is a promising novel way to combat cancer [12,17]. Moreover, in the case of ERK1/2 inhibition, where extensive studies have been carried out, the compensatory elevation in the ERK5 pathway has conferred resistance to the ERK1/2 therapy [77,78]. Development of an ERK5 inhibitor for a combination therapy with ERK1/2 inhibitors may contribute to overcoming this resistance [75,79]. In this study, we focused on confirming ERK5-inhibitory activity of MHJ-627 which was also identified in our in silico simulation model that MHJ-627 actually binds to an ATP-binding pocket of ERK5. In future studies, our next goal is to utilize MHJ-627 as a lead compound and modify it to be a more potent ERK5 inhibitor with increased specificity to ERK5 that can exhibit a powerful anticancer efficacy. Further study with improved ERK5 inhibitor will also include ERK5 knock-out and knock-down models to clearly demonstrate its specific ERK5-inhibitory efficacy.

## Figures and Tables

**Table 1:** List of primers used in quantitative real-time PCR analysis.

Gene	Primer Sequence (5′ to 3′)	References
*GAPDH*	F: GTGAAGGTCGGAGTCAACG R: TGAGGTCAATGAAGGGGTC	[37]
*PCNA*	F: AACCTCACCAGTATGTCCAA R: ACTTTCTCCTGGTTTGGTG	[40]
*DDIT4*	F: GTGGAGGTGGTTTGTGTATC R: CACCCCTTGCTACTCTTAC	This study
*CXCL1*	F: AAAGCTTGCCTCAATCCTGC R: CTTCAGGAACAGCCACCAGT	This study
*KLF4*	F: CCAATTACCCATCCTTCCTG R: CGATCGTCTTCCCCTCTTTG	This study
*NR4A1*	F: GCTTCATGCCAGCATTATGG R: GTTCGGACAACTTCCTTCAC	This study
*RORα*	F: AGGCTCGCTAGAGGTGGTGTT R: TGAGAGTCAAAGGCACGGC	This study
*PTPRC*	F: CTTCAGTGGTCCCATTGTGGTG R: CCACTTTGTTCTCGGCTTCCAG	This study
*CCL5*	F: TCATTGCTACTGCCCTCTGC R: TACTCCTTGATGTGGGCACG	This study
*ICAM1*	F: AGCGGCTGACGTGTGCAGTAAT R: TCTGAGACCTCTGGCTTCGTCA	This study
*SIGLEC1*	F: ACCTGGAGGAAACTGACAGTGG R: CTCAGTGTCACTGCCTGTCCTT	This study
*luc2P*	F: CTTTTGCAGCCCTTTCTTGC R: CTTTTGCAGCCCTTTCTTGC	This study

## Data Availability

All data generated or analyzed during this study are included in this published article.
